# Phlorotannin Extracts from Fucales Characterized by HPLC-DAD-ESI-MS*^n^*: Approaches to Hyaluronidase Inhibitory Capacity and Antioxidant Properties 

**DOI:** 10.3390/md10122766

**Published:** 2012-12-10

**Authors:** Federico Ferreres, Graciliana Lopes, Angel Gil-Izquierdo, Paula B. Andrade, Carla Sousa, Teresa Mouga, Patrícia Valentão

**Affiliations:** 1 Research Group on Quality, Safety and Bioactivity of Plant Foods, Department of Food Science and Technology, CEBAS (CSIC), P.O. Box 164, 30100 Campus University Espinardo, Murcia, Spain; E-Mail: angelgil@cebas.csic.es; 2 REQUIMTE/Laboratory of Pharmacognosy, Department of Chemistry, Faculty of Pharmacy, University of Porto, Rua de Jorge Viterbo Ferreira, no. 228, 4050-313 Porto, Portugal; E-Mails: gracilianalps@gmail.com (G.L.); pandrade@ff.up.pt (P.B.A.); csousa@ff.up.pt (C.S.); 3 GIRM—Marine Resources Research Group, School of Tourism and Maritime Technology, Polytechnic Institute of Leiria, Santuário N.ª Sra. Dos Remédios, Apartado 126, 2524-909 Peniche, Portugal; E-Mail: mougat@ipleiria.pt

**Keywords:** phlorotannins, skin aging, hyaluronidase, antioxidant activity, HPLC-DAD-ESI-MS*^n^*

## Abstract

Purified phlorotannin extracts from four brown seaweeds (*Cystoseira nodicaulis* (Withering) M. Roberts, *Cystoseira tamariscifolia* (Hudson) Papenfuss, *Cystoseira usneoides* (Linnaeus) M. Roberts and *Fucus spiralis* Linnaeus), were characterized by HPLC-DAD-ESI-MS*^n^*. Fucophloroethol, fucodiphloroethol, fucotriphloroethol, 7-phloroeckol, phlorofucofuroeckol and bieckol/dieckol were identified. The antioxidant activity and the hyaluronidase (HAase) inhibitory capacity exhibited by the extracts were also assessed. A correlation between the extracts activity and their chemical composition was established. *F. spiralis*, the species presenting higher molecular weight phlorotannins, generally displayed the strongest lipid peroxidation inhibitory activity (IC_50_ = 2.32 mg/mL dry weight) and the strongest HAase inhibitory capacity (IC_50_ = 0.73 mg/mL dry weight). As for superoxide radical scavenging, *C. nodicaulis* was the most efficient species (IC_50_ = 0.93 mg/mL dry weight), followed by *F. spiralis* (IC_50_ = 1.30 mg/mL dry weight). These results show that purified phlorotannin extracts have potent capabilities for preventing and slowing down the skin aging process, which is mainly associated with free radical damage and with the reduction of hyaluronic acid concentration, characteristic of the process.

## 1. Introduction

In the past few years, marine organisms have been studied by several scientific groups, mainly concerning the antioxidant, anti-inflammatory, anti-microbial and neuroprotective activity of their structurally diverse bioactive compounds [1,2,3,4,5,6]. Among these organisms, marine brown seaweeds play a significant role, as they are the only organisms on earth producing phlorotannins, which are polyphenols exhibiting important biological activities [2,7]. Phlorotannins are phloroglucinol-based compounds, biosynthesized by the acetate-malonate pathway, highly hydrophilic and with a wide range of molecular sizes [8]. They are classified into four main groups according to the linkages between phloroglucinol units: fuhalols and phloroethols (with ether linkages), fucols (with a phenyl linkage), fucophloroethols (with ether and phenyl linkages) and eckols (with dibenzodioxin linkages). Phloroethols differ from fuhalols by the presence of extra hydroxyl groups in the last [9]. In recent years, several phlorotannins have been isolated from seaweeds, allowing the knowledge of different structures. These polyphenols play a vital role in seaweeds, as they help to protect them from UV radiation, feeding by herbivores and oxidative stress originated from high oxygen concentrations that lead to the formation of free radicals and other strong oxidizing agents [7].

Seaweeds from the family Lessoniaceae (*Ecklonia* and *Eisenia* genus) and some members of Laminariaceae have been much studied for their phlorotannins’ biological activities. Among them, anti-bacterial, anti-allergy, anti-inflammatory and antioxidant activities are highlighted [2,4,5,6,9,10,11,12].

Reactive oxygen species (ROS), such as superoxide radical (O_2_^•−^), hydroxyl radical (HO^•^) and hydrogen peroxide (H_2_O_2_), formed during aerobic life, are recognized for being associated not only with initiation, but also with promotion and progression of multiple diseases, disorders and aging [13]. These ROS can target DNA and proteins to produce an array of toxic effects, such as peroxidation of lipid-rich membranes, leading to aberrant cell proliferation responses, which can exacerbate both allergic and inflammatory states [14]. The equilibrium between oxidants’ formation and endogenous antioxidant defense mechanisms should be maintained to protect cell biomolecules.

Skin is particularly vulnerable to ROS, since it is exposed to oxidative stress from both endogenous and exogenous sources [13,15]. Although oxidative stress is a key factor in this process, hyaluronic acid (HA) also plays a significant role [15]. This anionic, non-sulfated glycosaminoglycan forms the core of proteoglycan, which is responsible for maintaining the proper volume and flexibility of the skin. The integrity of HA inside the dermal matrix is essential for cell integrity, mobility and proliferation [13,16]. Under oxidative stress, hyaluronidase (HAase), an enzyme responsible for HA depolymerization, is over activated and excessively breaks down HA, leading to the destruction of the proteoglycan network. This results in the deregulation of skin homeostasis, aggravates inflammatory and allergic states and promotes the aged appearance of skin [16].

The effect of phlorotannins in preventing HA degradation via HAase inhibition is related not only to the prevention of skin aging, but also to the reduction of inflammatory states, allergy and migration of cancer cells. These compounds can effectively contribute to the recovery of skin homeostasis and consequently prevent the downstream events that physically damage dermal matrix structure. They are not only potent ROS scavengers, but have also demonstrated a huge capacity to inhibit HAase and to minimize the oxidative stress through a synergy created by the elimination of ROS and enhancement of the antioxidant defense capacity [11,17,18]. The non-toxic nature of these compounds should be valued, as they show an unparalleled low toxicity when compared with other natural antioxidants [19]. This feature, along with the potent anti-aging activities, is the hallmark of phlorotannins that enables effective protection from the loss of the skin elasticity of aged skin [16]. For these reasons, the use of natural anti-aging products derived from marine sources is gaining prominence and attracting researchers’ attention [3].

In this study, we demonstrate the anti-radical activity against superoxide radicals, the lipid peroxidation inhibitory capacity and the HAase inhibitory potential of four seaweed species belonging to the order Fucales (*Cystoseira nodicaulis* (Withering) M. Roberts, *Cystoseira tamariscifolia* (Hudson) Papenfuss, *Cystoseira usneoides* (Linnaeus) M. Roberts and *Fucus spiralis* Linnaeus). Phlorotannins of these species were also characterized by HPLC-DAD-ESI-MS*^n^*. To our knowledge, there is no report on phlorotannins characterization in *C. nodicaulis* and *C. usneoides*. Additionally, only fucols and fucophloroethols groups were identified in *F. spiralis* [20], and bifuhalol and diphloroethol were reported in *C. tamariscifolia* [21]*.* A correlation between these biological properties and the main identified phlorotannins, together with the potential pharmacological application of these extracts, is proposed.

## 2. Results and Discussion

Brown algae are characterized by the presence of a specific group of polyphenolic compounds, the phlorotannins [22]. These compounds possess chemical properties that enable their extraction and purification, allowing highly purified extracts to be obtained. The procedure starts with a pre-treatment with hexane to remove fats, followed by an extensive extraction with acetone:water (7:3), which is considered to be the most efficient solvent mixture to extract phlorotannins [22]. After extraction, a purification step takes place, in which the adherence capacity of phlorotannins to cellulose is used to separate them from undesirable co-extracted compounds. Pigments, including chlorophylls, are then easily removed by thoroughly washing cellulose with toluene, until the filtrate runs clear; afterwards, cellulose is rinsed with acetone:water (7:3) to release the phlorotannins [23]. 

In this work, the presence of phlorotannins in the purified extract was screened by the reaction with dimethoxybenzaldehyde, a reagent specific for 1,3- and 1,3,5-substituted phenols, which are characteristic of this class of compounds [24], before HPLC-DAD-ESI-MS*^n^* analysis. 

### 2.1. HPLC-DAD-ESI-MS*^n^* Phlorotannins Analysis

The Extracted Ion Chromatogram (EIC) of protonated molecular ions ([M + H]^+^) from the most common phlorotannins found in literature (dioxinodehydroeckol (371), eckol (373), fucophloroethol (375), 7-phloroeckol (497), fucodiphloroethol (499), phlorofucofuroeckol (603), fucotriphloroethol (623), dieckol (743), and fucophloroethols with six (747), seven (871) and eight units of phloroglucinol (995)) was used for the study of phlorotannins by HPLC-DAD-ESI-MS*^n^*. Furthermore, the MS fragmentations of other peaks observed in the UV chromatogram were studied.

**Figure 1:**
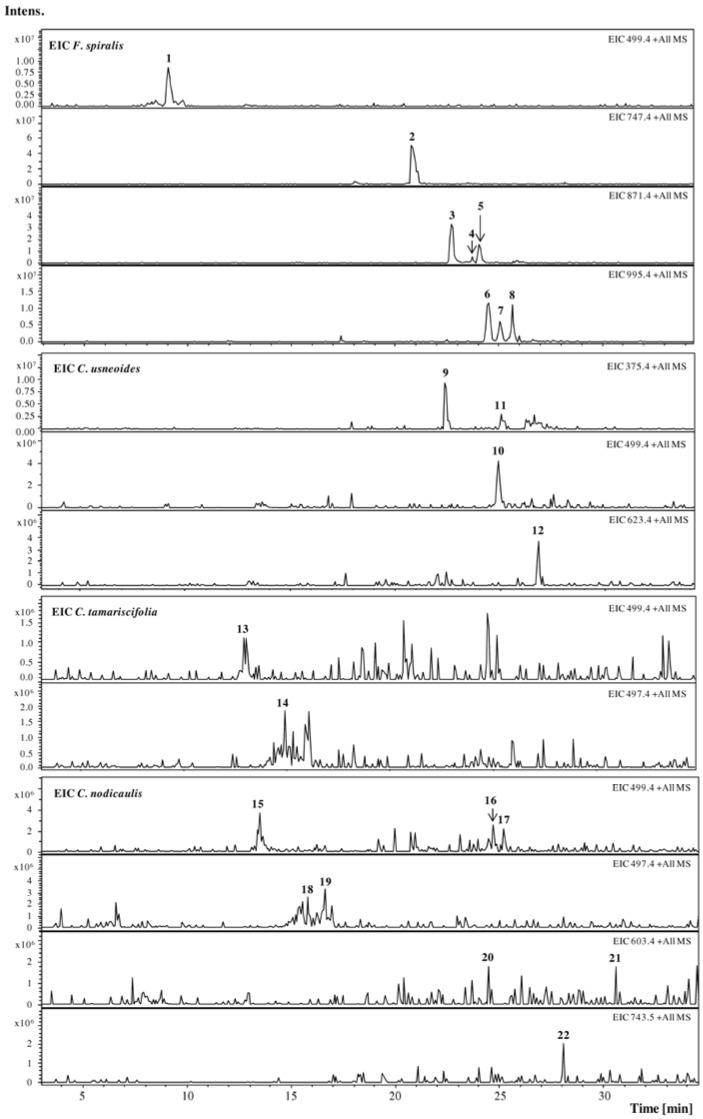
Extracted Ion Chromatogram (EIC) of purified phlorotannin extracts of *F. spiralis*, *C. usneoides*, *C. tamariscifolia* and *C. nodicaulis*. [M + H]^+^ (*m/z*): **1**, 499; **2**, 747; **3**–**5**, 871; **6**–**8**, 995; **9** and **11**, 375; **10**, 499; **12**, 623; **13**, 499; **14**, 497; **15**–**17**, 499; **18**–**19**, 497; **20**–**21**, 603; **22**, 743.

Phlorotannins analysis was carried out in *F. spiralis*, *C. usneoides*, *C. tamariscifolia* and *C. nodicaulis* purified extracts (Figure 1).

**Figure 2:**
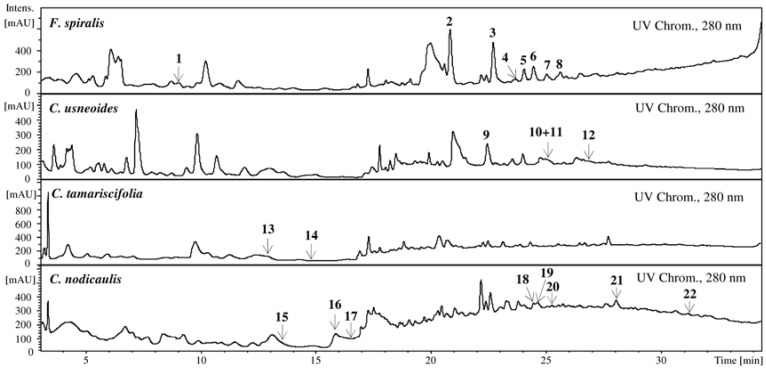
UV chromatograms of the purified phlorotannin extracts of *F. spiralis*, *C. usneoides*, *C. tamariscifolia* and *C. nodicaulis*, recorded at 280 nm. Identity of compounds as in Figure 1.

In the EIC of *F. spiralis* and of *C. usneoides*, the presence of well-defined and abundant ions, **1**–**8** and **9**–**12**, respectively, could be noticed, which can correspond tentatively to phlorotannins. On the other hand, in the EIC of *C. tamariscifolia* and *C. nodicaulis*, the ions are found in trace amounts and appear co-eluting with other compounds (Figure 1). Figure 2 shows the UV chromatograms recorded at 280 nm of the extracts of the four studied species, exhibiting some abundant peaks that do not correspond to any of the studied ions. Ions corresponding to the compounds tentatively identified as phlorotannins are not abundant in the chromatograms of *C. tamariscifolia* and *C. nodicaulis*.

**Figure 3:**
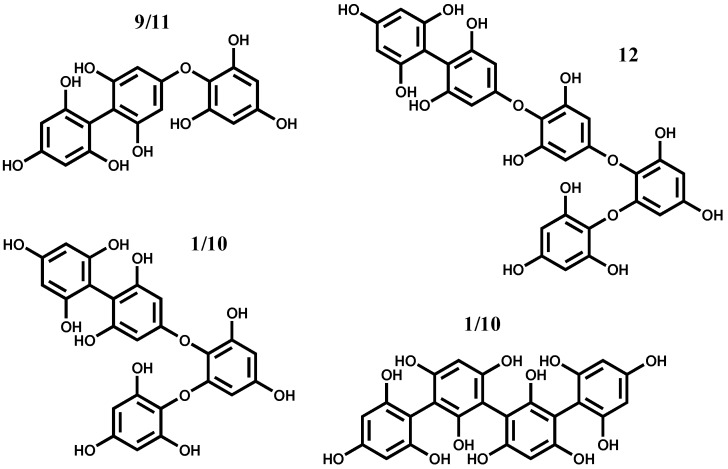
Hypothetical structure of the identified phlorotannins: Identity of compounds as in Figure 1.

**Figure 4:**
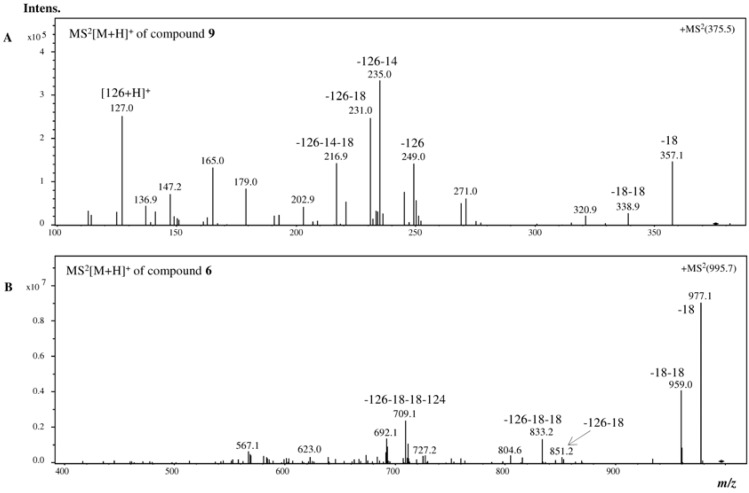
+MS^2^[M + H]^+^ analysis of (**A**) compound **9** from *C. usneoides* and (**B**) compound **6** from *F. spiralis*.

The MS study of the ions allowed the detection of two isomers, **9** and **11**, in *C. usneoides*, with protonated molecular ions ([M + H]^+^) at *m/z* 375 (Figure 3). These compounds showed similar fragmentation patterns in which some ions are characteristic of phlorotannins fragmentation, with losses of one and two molecules of water (−18 and −36, respectively), phloroglucinol (−126), as well as the protonated molecular ion of phloroglucinol (*m/z* 127), the base peak being the ion resulting from the loss of phloroglucinol and methyl (*m/z* 235, −126 − 14) (Figure 4A, Table 1). Based on the MS data, compounds **9** and **11** were considered to be phlorotannins composed by three phloroglucinol units and were tentatively identified as fucophloroethol isomers. In fact, isomers of fucophloroethols with the same molecular ion can occur, depending on the position of the aryl linkage [25].

Isomers with [M + H]^+^ at *m/z* 499, phlorotannins tetramers, compounds **1** in *F. spiralis* and **10** in *C. usneoides*, were also observed, which can correspond tentatively to tetrafucol and/or fucodiphloroethol (Figure 3). Their MS^2^ shows losses similar to those listed above (−18, −126/−127, among others), and the base peak in both is the ion at *m/z* 355 (−126 − 18). These losses were also detected in the other compounds, whose protonated molecular ions indicated them as polymers with several phloroglucinol units. Thus, in *C. usneoides*, compound **12** (with protonated molecular ion at *m/z* 623) is suggested to be a polyphenolic compound composed by five phloroglucinol units, possibly a fucotriphloroethol (Figure 3). In *F. spiralis*, compound **2** ([M + H]^+^ at *m/z* 747) can be a polyphenolic compound composed of six units of phloroglucinol.

Compounds **3**–**5**, with *m/z* 871, were tentatively identified as three isomers of phlorotannins. Although the peak corresponding to 871 mass units is routinely correlated with chlorophyll, the preparation of the extracts involved a purification step in order to remove pigments, namely chlorophylls, as referred above. Furthermore, the fragmentation pattern of the compounds, with specific mass losses consistent with phloroglucinol and water units (and no mass loss consistent with nitrogen, which exists in chlorophyll), led us to assume that the compounds are phlorotannins containing seven phloroglucinol units, which is in agreement with the literature [26]. The other three (compounds **6**–**8**) with *m/z* 995 (eight units of phloroglucinol) were also detected in this species (Figure 4B, Table 1).

**Table 1:** *R*t, UV and MS: [M + H]^+^, +MS^2^[M + H]^+^ data of compounds from *F. spiralis* (**1**–**8**), *C. usneoides* (**9**–**12**), *C. tamariscifolia* (**13**–**14**) and *C. nodicaulis* (**15**–**22**) ^a^.

	*R*t (min)	UV (nm)	[M + H]^+^, *m/z*	+MS^2^[M + H]^+^, *m/z* (%)
**1**	9.0	—	499	481 (80, −18), 439 (53, −18 − 42), 392 (25), 373 (35, −126), 355 (100, −126 − 18)
**2**	20.8	248, 268sh	747	729 (45, −18), 711 (70, −36), 585 (25, −126 − 36), 571 (35, −126 – 36 − 14), 220 (100)
**3**	22.7	244, 270sh	871	853 (100, −18), 835 (60, −36), 727 (30, −126 − 18), 709 (55, −126 − 36), 681 (30), 641 (12), 585 (15), 570 (7), 484 (35)
**4**	23.6	—	871	853 (100, −18), 835 (76, −36), 727 (38, −126 − 18), 709 (17, −126 − 36), 603 (22), 585 (20), 517 (9), 403 (7), 310 (4)
**5**	24.0	274sh	871	853 (67, −18), 835 (100, −36), 725 (25), 709 (55, −126 − 36), 694 (58), 681 (21), 569 (17), 551 (18), 435 (20)
**6**	24.4	272sh	995	977 (100, −18), 959 (45, −36), 851 (3, −126 − 18), 833 (15, −126 − 36), 709 (26, −126 − 124 − 36), 692 (15)
**7**	25.0	274sh	995	977 (100, −18), 959 (27, −36), 869 (7, −126), 851 (8, −126 − 18), 833 (12, −126 − 36), 709 (9, −126 − 124 − 36), 692 (6)
**8**	25.6	274sh	995	977 (100, −18), 959 (8, −36), 851 (19, −126 − 18), 833 (14, −126 − 36), 709 (12, −126 − 124 − 36), 692 (14)
**9**	22.5	270	375	357 (44, −18), 339 (2, −36), 249 (42, −126), 235 (100, −126 − 14), 231 (74, −126 − 18), 217 (43), 127 (75, floroglc + H)
**10**	25.0	—	499	481 (80, −18), 453 (18, −18 − 28), 419 (70), 372 (33, −127), 359 (30, −126 − 14), 355 (100, −126 − 18), 301 (42), 179 (43)
**11**	25.2	—	375	357 (23, −18), 339 (7, −36), 249 (40, −126), 235 (100, −126 − 14), 317 (33), 127 (57, floroglc + H)
**12**	26.8	—	623	605 (33, −18), 577 (18, −18 − 28), 523 (50), 497 (2, −126), 483 (12, −126 − 14), 479 (10, −126 − 18), 337 (44), 231 (100)
**13**	13.0	—	499	481 (49, −18), 411 (29), 397 (28), 395 (25), 359 (25, −162 − 14), 356 (17), 250 (16), 231 (100), 166 (33)
**14**	14.9	—	497	479 (9, −18), 462 (24), 451 (30, −18 − 28), 386 (48), 368 (70), 351 (34), 258 (100), 240 (38), 222 (37)
**15**	13.5	—	499	481 (70, −18), 373 (24, −126), 355 (70, −126 − 18), 341 (52, −126 − 18 − 14), 291 (33), 272 (70), 249 (100)
**16**	15.8	—	497	479 (100, −18), 463 (24), 451 (31, −18 − 28), 368 (8), 351 (45), 298 (14), 258 (41)
**17**	16.7	—	497	479 (83, −18), 462 (100), 452 (8), 435 (18), 385 (32), 368 (74), 351 (23), 258 (53)
**18**	24.5	—	603	559 (100, −44), 482 (94), 464 (78)
**19**	24.7	—	499	481 (100, −18), 368 (38), 247 (25), 242 (25)
**20**	25.2	—	499	462 (9), 368(100), 334 (12)
**21**	28.1	—	743	725 (45, −18), 715 (80, −28), 701 (97, −42), 685 (10), 633 (100), 600 (33)
**22**	30.5	—	603	585 (100, −18), 523 (15), 458 (17), 368 (56), 301 (30)

^a^ Main observed fragments. Other ions were found, but they have not been included.

Species belonging to the order Fucales [26,27] and Laminariales [12,28] have been the subject of many studies, allowing the identification of several phlorotannins structures, such as eckol, dieckol, 8,8′ dieckol, 6,6′ bieckol, 8,8′ bieckol, phlorofucofuroeckol-A and B, fucofuroeckol-A, dioxinodehydroeckol, 2-phloroeckol, 7-phloroeckol, fucodiphloroethol-G and triphloroethol. 

Phlorotannins elucidation is a rather complex task, even more so as no reference compounds are commercially available. Recently, Steevensz and his research group characterized the phlorotannins of five brown algae species by ultrahigh-pressure liquid chromatography operating in hydrophilic interaction liquid chromatography mode and combined with high resolution mass spectrometry [25]. The methodology proposed by this research group proved to be accurate for profiling phlorotannins based on their degree of polymerization, but did not allow the identification of the phlorotannins’ chemical groups.

Thus, in the present work, 22 different phlorotannins belonging to eckol and fucophloroethol main groups were characterized in the studied seaweeds: eight in *C. nodicaulis*, two in *C. tamariscifolia*, four in *C. usneoides* and eight in *F. spiralis* (Figure 1 and Figure 2, Table 1). To our knowledge, with the exception of fucols and fucophloroethols groups identified in *F*. spiralis [20], and bifuhalol and diphloroethol identified in *C. tamariscifolia* [21], none of the compounds described herein were previously reported in these species.

### 2.2. Antioxidant Activity

**Figure 5:**
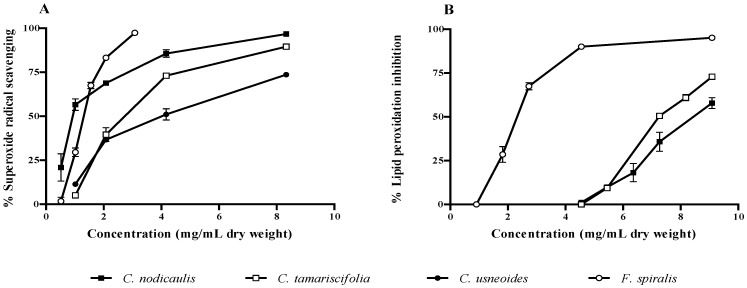
Activity of purified phlorotannin extracts against superoxide radical (**A**) and lipid peroxidation (**B**). Results are expressed as percentage relative to control (mean ± standard deviation of three independent assays).

The antioxidant potential of purified phlorotannin extracts was checked against superoxide radicals and lipid peroxidation. A concentration-dependent pattern was observed in both assays. *C. nodicaulis* and *F. spiralis* were the species with the highest radical scavenging capacity, presenting IC_50_ values of 0.93 and 1.30 mg/mL (dry weight), respectively (Figure 5A, Table 2). The IC_50_ found for these two species was significantly lower than those obtained for *C. tamariscifolia* and *C. usneoides* (*P* < 0.0001). Concerning lipid peroxidation inhibition, *F. spiralis* was the most effective, displaying an IC_50_ value (2.32 mg/mL, dry weight) significantly lower than those found for both *C. nodicaulis* and *C. tamariscifolia* (*P* < 0.0001) (Figure 5B, Table 2). The protective activity of *C. usneoides* against lipid peroxidation was not higher than 15% for the highest concentration tested (100 mg/mL dry weight, Table 2).

**Table 2:** Biological activity of purified phlorotannin extracts ^a^.

Samples	Assays		
	Superoxide radical scavenging	Lipid peroxidation inhibition	HAase inhibition				
***C. tamariscifolia***	2.73 (0.30)	7.55 (0.02)	3.3 (0.12)
***C. usneoides***	4.02 (0.68)	>9.10	2.51 (0.22)
***F. spiralis***	1.30 (0.04)	2.32 (0.08)	0.73 (0.06)
***C. nodicaulis***	0.93 (0.11)	8.52 (0.08)	1.34 (0.04)

^a^ IC_50_ values (mg/mL, dry weight), represented as mean (standard deviation) of three assays, performed in triplicate.

Anti-radical activity is highly important when there is an uncontrolled production of reactive species in the body and the endogenous antioxidants are not able to overcome their deleterious effects. Oxidative stress occurs in the cells because an imbalance between the pro-oxidant/antioxidant systems takes place, causing injury to biomolecules, such as nucleic acids, proteins, structural carbohydrates and lipids. Among these targets, the peroxidation of lipids is basically damaging, because the derived products lead to the spread of free radical reactions [13,14].

Skin aging has been associated with the increased peroxidation of skin lipids, namely of squalene, which constitutes the main component of skin surface polyunsaturated lipids and is easily peroxided. Peroxidation of fatty acyl groups of membrane phospholipids is one of the major outcomes of free radical mediated injury to tissue, leading to cell damage by continuous chain reactions. In addition, lipid peroxidation may be responsible for the activation of phospholipase A2, production of many mediators by arachidonate, deactivation of adenylate cyclase and activation of guanilate cyclase, leading to the decrease of the cAMP/cGMP ratio, responsible for epidermal proliferation. Thus, the physicochemical properties of the membrane lipid bilayers can be greatly altered, resulting in severe cellular dysfunction [14].

Several works have concerned the antioxidant activity of crude phlorotannin extracts and isolated compounds. Zubia and co-workers assessed the antioxidant capacity of 10 brown seaweeds from the Brittany coast [29]. Species belonging to the order Fucales, mainly the ones of the genus *Fucus* and *Cystoseira*, displayed the strongest activity, which was positively correlated with their phenolics content. Audibert *et al.* studied the radical scavenging activity of purified extracts of phlorotannins against DPPH and ABTS radicals and concluded that the antioxidant activity of the extracts was related to the content of phlorotannins and to their molecular weight [5]. Similar results were obtained by Shibata and colleagues with phlorotannins isolated from Laminariaceous [12]. These authors found that the increase of molecular weight of the isolated phlorotannins leads to a decrease of the antioxidant activity. On the other hand, Wang *et al.* assessed the antioxidant activity of phlorotannins fractions from *Fucus vesiculosus* Linnaeus against DPPH radical, but no clear relationship was found between the antioxidant capacity and the degree of polymerization/molecular weight of phlorotannins [4]. Liu and Gu studied the ability of the same species to scavenge carbonyls by the formation of adducts, concluding that phlorotannins were effective in inhibiting the formation of advanced glycation end products, which are a class of compounds associated with skin aging [6].

According to these previous reports, it is possible to establish a correlation between phlorotannins composition and biological activity. Thus, *F. spiralis* is the species displaying the lowest IC_50_ regarding lipid peroxidation inhibition and *C. nodicaulis* the one showing the best superoxide scavenging capacity, but almost no activity against lipid peroxidation (Figure 4, Table 2). It seems that extracts with lower molecular weight phlorotannins are more effective as superoxide radical scavengers, and those with higher molecular weight are better in inhibiting lipid peroxidation (Table 1 and Table 2).

Recently, we have reported the antioxidant activity of purified phlorotannin extracts, addressed by the nitric oxide (^•^NO) scavenging ability, in cell free systems. *F. spiralis* and *C. nodicaulis* exhibited the highest capacity for ^•^NO scavenging [2]. It is well known that ^•^NO reacts rapidly with superoxide radical to form peroxynitrite (ONOO^−^) [30]. As the studied species showed capacity for scavenging both ^•^NO and superoxide radicals, they can prevent the generation of ONOO^−^, a strong oxidant with a lethal effect on many cells.

### 2.3. Inhibitory Effect of Brown Algae Phlorotannins on HAase

**Figure 6:**
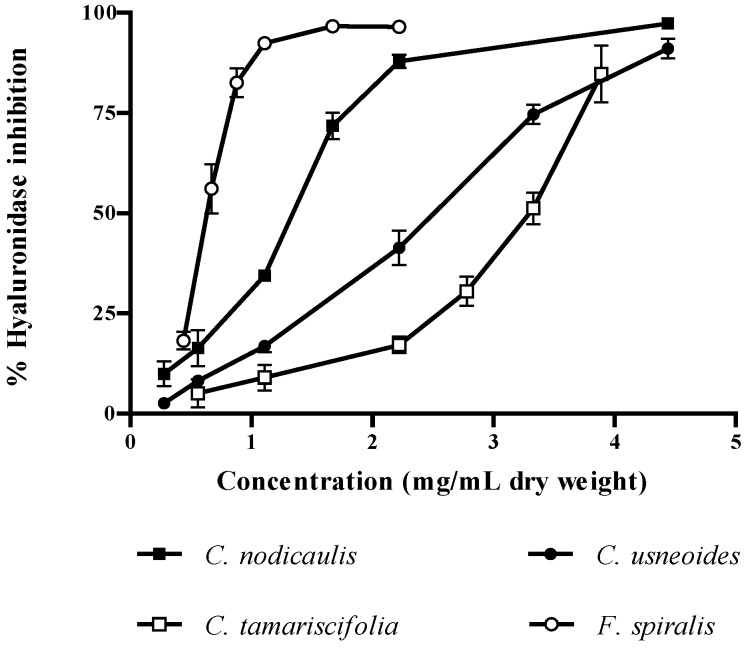
Inhibitory activity of purified phlorotannin extracts against hyaluronidase. Results are expressed as percentage of hyaluronidase inhibition relative to control (mean ± standard deviation of three independent assays).

As referred to above, HAase is an enzyme responsible for the depolymerization of HA, a polysaccharide present in the extracellular matrix of the connective tissue and in body fluids. This enzyme is known to be involved in allergic effects, migration of cancer and inflammation, increasing the permeability of the vascular system. As so, potent HAase inhibitors might have anti-allergic, anticancer and anti-aging activities, becoming leading compounds in the development of new drugs [7,15]. Of the species studied herein, *F. spiralis* demonstrated the highest capacity for HAase inhibition (IC_50_ = 0.73 mg/mL dry weight), *C. tamariscifolia* being the less effective (Figure 6, Table 2). All of the IC_50_ values found were significantly different (*P* < 0.0001).

Phlorotannins are reported as anti-allergic and potential anti-aging agents, owing to their strong inhibitory effect on the activation of HAase. Sugiura and co-workers performed a bioguided fractionation of a methanol:chloroform extract of the brown seaweed *Eisenia arborea* Areschoug and identified six phlorotannins, phlorofucofuroeckol-B showing the strongest activity [31]. HAase inhibition assay was also performed with crude phlorotannins extracted from five brown seaweeds from Pakistan and China [10]. A positive correlation between the extracts activity and the total phlorotannins content was observed. *Sargassum tennerimum* J. Agardh was the most active species, exhibiting an IC_50_ value lower than that of disodium cromoglycate, a natural inhibitor of hyaluronidase. In addition, Shibata and colleagues observed that phlorotannins, like eckol, phlorofucofuroeckol A, dieckol and 8,8′-bieckol, exert a stronger inhibition of hyaluronidase than well-known inhibitors, such as catechin and sodium cromoglycate, and that phlorotannins with higher molecular weight are more effective [11].

Our findings seem to be in good agreement with previous reports. In fact, the better results obtained with *F. spiralis* extract are not surprising, as this species possesses the highest total phlorotannins content [2]. Furthermore, it also contains the ones with higher molecular weight, as demonstrated herein (Figure 2, Table 1). However, the structure of phlorotannins appears to be more important than its amount. The genus *Cystoseira* is a good example: despite having a much lower phlorotannins content than *C. tamariscifolia* [2], *C. nodicaulis* and *C. usneoides* present significantly lower IC_50_ values for HAase inhibition (Figure 6, Table 2).

## 3. Experimental Section

### 3.1. Standards and Reagents

Formic acid, sodium chloride (NaCl), HAase from bovine testes (type IV-S), β-nicotinamide adenine dinucleotide reduced form (NADH), nitrotetrazolium blue chloride (NBT), phenazine methosulphate (PMS), L-ascorbic acid, sodium formate, trizma hydrochloride (Tris-HCl) and bovine serum albumin (BSA) were from Sigma-Aldrich (Steinheim, Germany). HA sodium salt from *Streptococus equi* was from Sigma-Aldrich (Prague, Czech Republic). Linoleic acid was from Calbiochem (San Diego, USA). Ethanol, hydrochloric acid (HCl), diethyl ether and potassium hydroxide (KOH) were from Panreac (Barcelona, Spain). HPLC-grade methanol, acetonitrile, potassium di-hydrogen phosphate buffer, di-sodium tetraborate, iron (II) sulfate (FeSO_4_·7H_2_O) and 4-dimethylaminobenzaldehyde (DMAB) were obtained from Merck (Darmstadt, Germany). Glacial acetic acid was purchased from Fisher Scientific (Loughborough, UK). 

Water was deionized using a Milli-Q water purification system (Millipore, Bedford, MA, USA).

### 3.2. Samples

Brown seaweeds used in this work are indigenous from the coast of Peniche (West Portugal). Several individuals in the same stage of development were randomly collected and identified by Teresa Mouga, PhD (GIRM). *C. usneoides* and *C. nodicaulis* were collected in 2009 and *C. tamariscifolia* and *F. spiralis* were collected in 2008. 

After collection, samples were immediately transported to the laboratory in ice boxes, washed with sea water, frozen and lyophilized in a Labconco 4.5 Freezone apparatus (Kansas City, MO, USA). Thereafter, the dried samples were ground (particle size ≤910 µm) and stored into a desiccator, in the dark, until phlorotannins extraction.

### 3.3. Extraction and Purification

Dried purified phlorotannin extracts were obtained as reported before [2]. Briefly, the powdered material was defatted with hexane and extracted with acetone:water (7:3). The extract was purified with cellulose, which was further washed with toluene. Afterwards, cellulose was rinsed with acetone:water (7:3) to recover the adhered phlorotannins, the filtrate was evaporated and the dried extract was dissolved in the appropriate solvent prior to analysis. 

### 3.4. HPLC-DAD-ESI-MS*^n^* Analyses

Chromatographic analyses were carried out on a Luna C18 column (250 × 4.6 mm, 5 µm particle size; Phenomenex, Macclesfield, UK). The mobile phase consisted of two solvents: 1% formic acid in water (A) and acetonitrile (B). Elution was performed as follows: 0–10 min, 0% B; 30 min, 30% B; 35 min, 80% B; 40 min, 80% B; 42 min, 0% B; 52 min, 0% B. The flow rate was 1 mL/min and the injection volume was 20 µL. Spectral data from all peaks were accumulated in the range 240–400 nm, and chromatograms were recorded at 280 nm. The HPLC-DAD-ESI-MS*^n^* (*n* = 1–2) analyses were carried out in an Agilent HPLC 1100 series equipped with a diode array detector and mass detector in series (Agilent Technologies, Waldbronn, Germany). The HPLC consisted of a binary pump (model G1312A), an auto-sampler (model G1313A), a degasser (model G1322A) and a photodiode array detector (model G1315B). The HPLC system was controlled by the ChemStation software (Agilent, v. 08.03). The mass detector was an ion trap spectrometer (model G2445A) equipped with an electrospray ionization interface and was controlled by LCMSD software (Agilent, v. 4.1). The ionization conditions were adjusted at 350 °C and 4 kV for capillary temperature and voltage, respectively. The nebulizer pressure and flow rate of nitrogen were 65.0 psi and 11 L/min, respectively. The full scan mass covered the range from *m/z* 100 up to *m/z* 1500. Collision-induced fragmentation experiments were performed in the ion trap using helium as the collision gas, with voltage ramping cycles from 0.3 up to 2 V. Mass spectrometry data were acquired in the positive ionization mode.

In Figure 2 and Table 1, the compounds were numbered following elution order. As so, numbering started by *F. spiralis* (**1**–**8**) and followed to *C. usneoides* (**9**–**12**), *C. tamariscifolia* (**13**, **14**) and *C. nodicaulis* (**15**–**22**).

### 3.5. Antioxidant Activity

#### 3.5.1. Superoxide Radical Scavenging Assay

The methodology for evaluating the superoxide radical scavenging activity was described by Oliveira and co-workers [32]. Briefly, serial dilutions of purified phlorotannin extracts were prepared. Superoxide radical was generated by the NADH/PMS system. The scavenging activity was determined by monitoring the effect on the reduction of NBT induced by superoxide radicals, in the presence and absence of purified phlorotannin extracts, using a Multiscan Ascent plate reader (Thermo Electron Corporation) working in kinetic function, at 562 nm. All components were dissolved in phosphate buffer (19 mM, pH 7.4). Three independent assays were performed in triplicate.

#### 3.5.2. Lipid Peroxidation Inhibition Assay

Peroxidation of fatty acyl groups was determined following the methodology proposed by Choi and co-workers [33] and Shimasaki [34], with slight modifications. The reaction mixture contained 250 µL of linoleic acid (20 mM in ethanol), 150 µL of Tris-HCl (100 mM, pH 7.5), 50 µL of FeSO_4_·7H_2_O (4 mM in water) and 50 µL of serial dilutions of purified phlorotannin extracts prepared in distilled H_2_O. Linoleic acid peroxidation was initiated by the addition of 50 µL of ascorbic acid (5 mM in water), and the mixture was immediately incubated for 1 h at 37 °C. After the incubation period, 1.5 mL of ethanol:ether (3:1) mixture was added to each test tube, to allow the rearrangement of double bonds, which results in the formation of conjugated dienes (lipid hydroperoxides). The mixtures were vortexed and the absorbance was immediately measured at 233 nm in a Helios α (Unicam) spectrophotometer, at room temperature. Three independent assays were performed in triplicate.

### 3.6. HAase Inhibition Assay

HAase inhibition assay was modified from that proposed by Muckenschnabela and co-workers [35]. A stock solution of 5 mg/mL HA was prepared in water and stored at 4 °C. HA stock solution (50 µL) and 100 µL of buffer (0.2 M sodium formate, 0.1 M NaCl and 0.2 mg/mL BSA, pH adjusted to 3.68 with formic acid) were added to 200 µL of water. Sample serial dilutions were prepared in water and 50 µL of each dilution were added to each reaction tube. The reaction was started by the addition of 50 µL of HAase (600 U/mL) prepared in NaCl 0.9%.

The enzymatic reaction was stopped by adding 25 µL of an alkaline solution consisting of di-sodium tetraborate (0.8 M in water) and subsequent heating for 3 min in a boiling water bath. The test tubes were cooled at room temperature and 750 µL of DMAB solution was added (2 g of DMAB dissolved in a mixture of 2.5 mL of 10 N HCl and 17.5 mL of glacial acetic acid and further diluted 1:2 with glacial acetic acid immediately before use). 

The tubes were incubated at 37 °C for 20 min. The absorbance of the colored product was measured at 560 nm in a Multiskan Ascent plate reader. Three independent assays were performed in triplicate.

### 3.7. Statistical Analysis

Data were analyzed by using GraphPad PRISM software (GraphPad software, San Diego, CA, USA) (version 5.02 for Windows). One-way analysis of variance (ANOVA), using the Turkey’s multiple comparison test, was carried out on data obtained from triplicate determinations of each sample. A level of statistical significance at *P* < 0.05 was used.

## 4. Conclusions

The present study showed that purified phlorotannin extracts inhibited hyaluronidase activity and presented a good radical scavenging activity. Our study opens up a new line of research on the antioxidant and anti-hyaluronidase activity of seaweed phlorotannins, especially in relation to their anti-aging potential. Species like *F. spiralis* and *C. nodicaulis* are promising candidates for the development of new pharmaceutical formulas with the capacity to protect skin cells from oxidative damage, slowing down skin aging by preventing the age-dependent loss of hyaluronic acid content and accelerating its recovery in inflammatory and allergic states. Moreover, the analysis of the extracts by HPLC-DAD-ESI-MS*^n^* improved knowledge on the phlorotannins composition of seaweeds. Fucodiphloroethol and 7-phloroeckol were identified in *C. tamariscifolia* for the first time, as fucodiphloroethol was in *F. spiralis*. As far as we know, this is the first report on *C. usneoides* and *C. nodicaulis* phlorotannins characterization.
